# Functional characterization of the meiosis-specific DNA double-strand break inducing factor SPO-11 from *C. elegans*

**DOI:** 10.1038/s41598-017-02641-z

**Published:** 2017-05-24

**Authors:** Hsin-Yi Yeh, Sheng-Wei Lin, Yi-Chun Wu, Nei-Li Chan, Peter Chi

**Affiliations:** 1 Institute of Biochemical Sciences, National Taiwan University, NO. 1, Section 4, Roosevelt Road, Taipei, 10617 Taiwan; 2 Institute of Biological Chemistry, Academia Sinica, 128 Academia Road, Section 2, Nankang, Taipei 11529 Taiwan; 3 Institute of Molecular and Cellular Biology and Department of Life Science, National Taiwan University, NO. 1, Section 4, Roosevelt Road, Taipei, 10617 Taiwan; 4 Institute of Biochemistry and Molecular Biology, College of Medicine, National Taiwan University, NO. 1, Sec.1, Ren-Ai Rd., 100 Taipei, Taiwan

## Abstract

The programmed induction of meiotic DNA double-strand breaks (DSBs) by the evolutionarily conserved SPO-11 protein, which is structurally related to archaeal Topo VIA topoisomerases, triggers meiotic recombination. Identification of several meiosis-specific factors that are required for SPO-11-mediated DSB formation raises the question whether SPO-11 alone can cleave DNA. Here, we have developed procedures to express and purify *C. elegans* SPO-11 in a soluble, untagged, and monodispersed form. Our biochemical and biophysical analyses demonstrate that SPO-11 is monomeric and binds DNA, double-stranded DNA in particular. Importantly, SPO-11 does not exhibit DNA cleavage activity under a wide range of reaction conditions, suggesting that co-factors are needed for DSB induction activity. Our SPO-11 purification system and the findings reported herein should facilitate future mechanistic studies directed at delineating the mechanism of action of the SPO-11 ensemble in meiotic DSB formation.

## Introduction

Meiotic recombination helps establish physical connections between homologous chromosomes, which helps ensure proper chromosome segregation in the first meiotic division and also serves to reshuffle genetic information that generates genetic diversity^1–3^. As such, dysregulation of meiotic recombination results in chromosome non-disjunction and sterility. Meiotic recombination is a tightly regulated process that is triggered by the programmed induction of DNA double-strand breaks (DSBs)^1–4^. Once formed, the ends of the DSBs are nucleolytically processed to generate 3′ single-stranded DNA (ssDNA) tails. Meiotic recombination factors then engage these ssDNA tails to form a nucleoprotein ensemble capable of locating DNA homology in the chromosome homologue and mediating invasion of the homologue to form a DNA joint called a displacement loop or D-loop. The 3′ end of the invading strand is extended by DNA synthesis, followed by the pairing of the non-invading 3′ single-stranded tail with the displaced ssDNA strand in the enlarged D-loop (second end capture). After DNA synthesis and DNA ligation, a double Holliday Junction (dHJ) intermediate is formed. Resolution of the dHJ intermediate can result in crossover recombinants that harbor a reciprocal exchange of the arms of the homologous chromosomes^1, 2, 4–6^.

Genetic studies have revealed that meiotic DSBs arise via the action of a protein ensemble that harbors the Spo11 protein, which bears homologous to archaeal Topo VIA, the catalytic subunit of a type II topoisomerase^4, 7–10^. Indeed, studies in *S. cerevisiae*, *S. pombe*, and *M. musculus* have shown that Spo11 becomes covalently conjugated to the 5′ ends of DNA through a tyrosine residue proposed to be the catalytic center of topoisomerase function^11–14^. Thus, mutations in the putative catalytic tyrosine residue of Spo11 engender the same phenotype as *spo11* deletion in *S. cerevisiae* ^8, 9^, *S. pombe* ^15^, *A. thaliana* ^16^ and *M. musculus* ^17^. All these observations suggest that Spo11 is directly involved in catalyzing DSB formation to trigger meiotic recombination. Consistent with this premise, the *C. elegans spo-11* null mutant is unable to conduct meiotic recombination and, as a consequence, fails to generate crossovers and chiasmata^18^. Importantly, the introduction of radiation-induced DSBs partially alleviates the phenotype of mutant animals, thus emphasizing the key role of SPO-11 in meiotic DSB genesis^18^.

Genetic studies in various model organisms have identified several meiosis-specific factors that are required for SPO-11-mediated DSB formation^3, 4, 12, 19–21^. Notably, a Topo VIB-like subunit has recently been identified in plant and mouse and functions with SPO11 for the DSB formation^4, 22, 23^. The discovery of these accessory cofactors invites further investigation into whether SPO-11 protein alone possesses the ability to cleave DNA or if these other factors are needed for activating DNA cleavage by SPO-11. To date, efforts in delineating the biochemical properties of SPO-11 have been hindered by the challenge of obtaining soluble recombinant protein for functional analyses. Here, we have established procedures to express and purify *C. elegans* SPO-11 in a soluble, monodispersed form. Our functional analyses demonstrate that SPO-11 binds linear and supercoiled duplex DNA species with a higher affinity than single-stranded DNA. Biochemical and biophysical analyses reveal that SPO-11 is monomeric, which highlights a major difference with its homodimeric archaeal Topo VIA counterpart^7, 24–26^. Importantly, SPO-11 has no DNA cleavage activity under a variety of biochemical conditions. These results suggest that activation of the topoisomerase function of SPO-11 requires the action of cofactors. The protein expression and purification procedures that we have developed should facilitate the identification of these co-factors.

## Results

### Expression and purification of *C. elegans* SPO-11 protein

**Figure 1:**
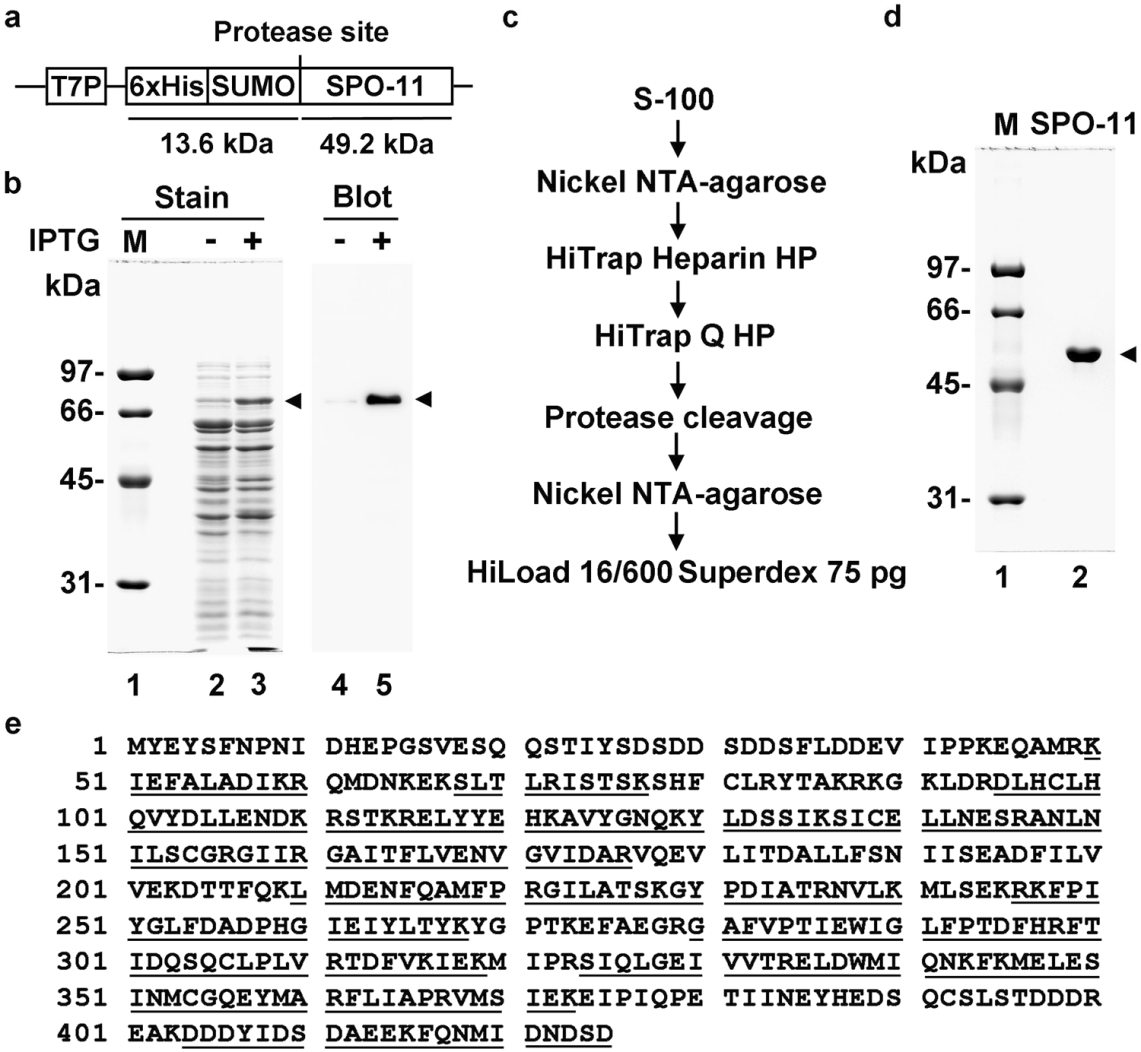
Expression and purification of *C. elegans* SPO-11. (**a**) The SPO-11 expression vector. (**b**) Extracts from *E. coli* cells harboring the SPO-11 expression plasmid grown with or without IPTG were analyzed by 10% SDS-PAGE and stained with Coomassie Blue or Western blot analysis with anti-His antibody. (**c**) Flow chart of SPO-11 purification. (**d**) Purified SPO-11 (1.5 μg) was analyzed by SDS-PAGE. (**e**) Results from MALDI-TOF analysis of purified SPO-11. Identified fragments are underlined.

The biochemical characterization of SPO-11 has been hampered by the difficulty in obtaining soluble, monodispersed preparations of the protein. To help overcome this hurdle, we introduced N-terminal six histidine (His)_6_ and SUMO tags into *C. elegans SPO-11* cDNA and expressed the recombinant protein in *E. coli* (Fig. 1a). The SUMO tag enhances protein solubility but can be easily removed with the (His)_6_ tag by treatment with the SUMO protease Ulp1. The T7 promoter that is inducible by isopropyl β-D-1-thiogalactopyranoside (IPTG) drives the robust expression of the tagged SPO-11 protein (Fig. 1b). The IPTG-inducible expression of the tagged protein was verified by Western blot analysis using anti-His antibody (Fig. 1b). We devised a purification scheme that entails affinity, ion-exchange, and gel filtration chromatographic steps to purify SPO-11 to near homogeneity (Fig. 1c and d); note that the (His)_6_ and SUMO tags are removed through Ulp1 protease treatment during purification. The purified, untagged SPO-11 protein (calculated molecular mass of 49,169 Da) migrates in SDS-PAGE analysis as a ~55 kDa species (Fig. 1d), and its identity was established by liquid chromatography-tandem mass spectrometry (LC-MS/MS) (Fig. 1e). Several independent preparations of SPO-11 gave the same results in all the biochemical and biophysical experiments described below.

### Monomeric nature of SPO-11

**Figure 2:**
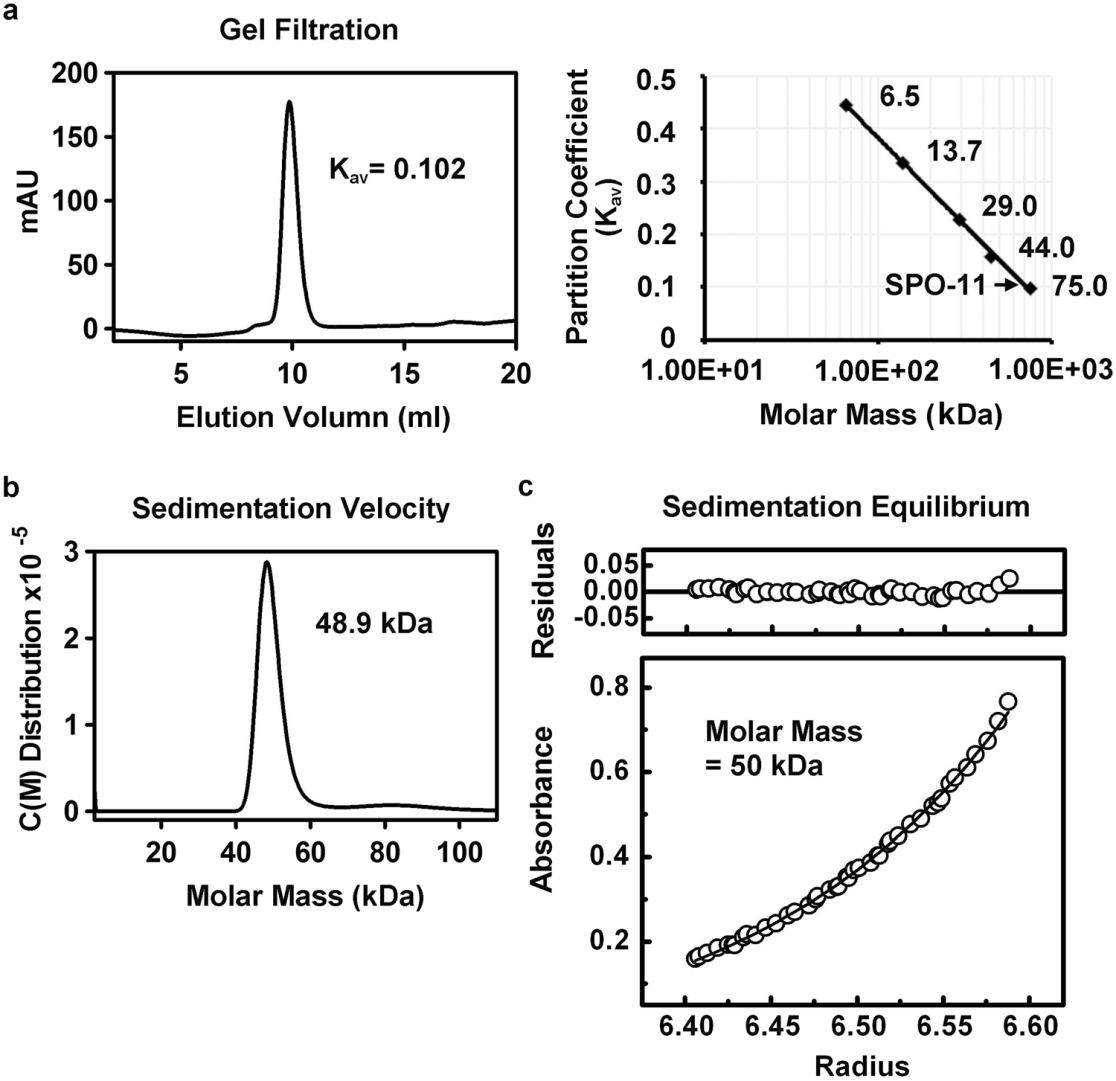
*C. elegans* SPO-11 is monomeric in solution. (**a**) Purified SPO-11 was analyzed in a Superdex 75 10/300 GL column. A plot of the partition coefficient (K_av_) versus molar mass with size standards was used to calculate the apparent molecular weight of SPO-11. (**b**) Analytical ultracentrifugation (AUC) with sedimentation velocity analysis of SPO-11. The experimental data were analyzed by the Sedfit program (version 12.1), which yielded an estimated molecular mass of 48.9 kDa. (**c**) Sedimentation equilibrium analysis of recombinant SPO-11. The average of molecular mass was estimated to be 50 kDa. The upper part of the figure shows the residual difference between experimental and fitted values by the standard deviation.

The oligomeric status of purified SPO-11 was assessed by size-exclusion analysis in Superdex 75. The elution profile of untagged SPO-11 revealed a size of ~70 kDa in correspondence to the size standards (Fig. 2a), suggesting a monomeric nature of this protein. Analytical ultracentrifugation (AUC) experiments with sedimentation velocity and equilibrium methods were then conducted to further ascertain the monomeric nature of SPO-11. Velocity ultracentrifugation revealed an apparent 48.9 kDa molecular mass of SPO-11 (Fig. 2b). In the equilibrium analysis, the concentration distribution of protein species depends only on their molecular mass. The sedimentation profile of SPO-11 showed a molecular mass of 50 kDa (Fig. 2c). The above analyses provided excellent concordance with the calculated molecular weight of 49.2 kDa for the monomeric protein. The biophysical results thus indicate that *C. elegans* SPO-11 is a monomer in solution.

### SPO-11 possesses thermal labile DNA-binding activity

**Figure 3:**
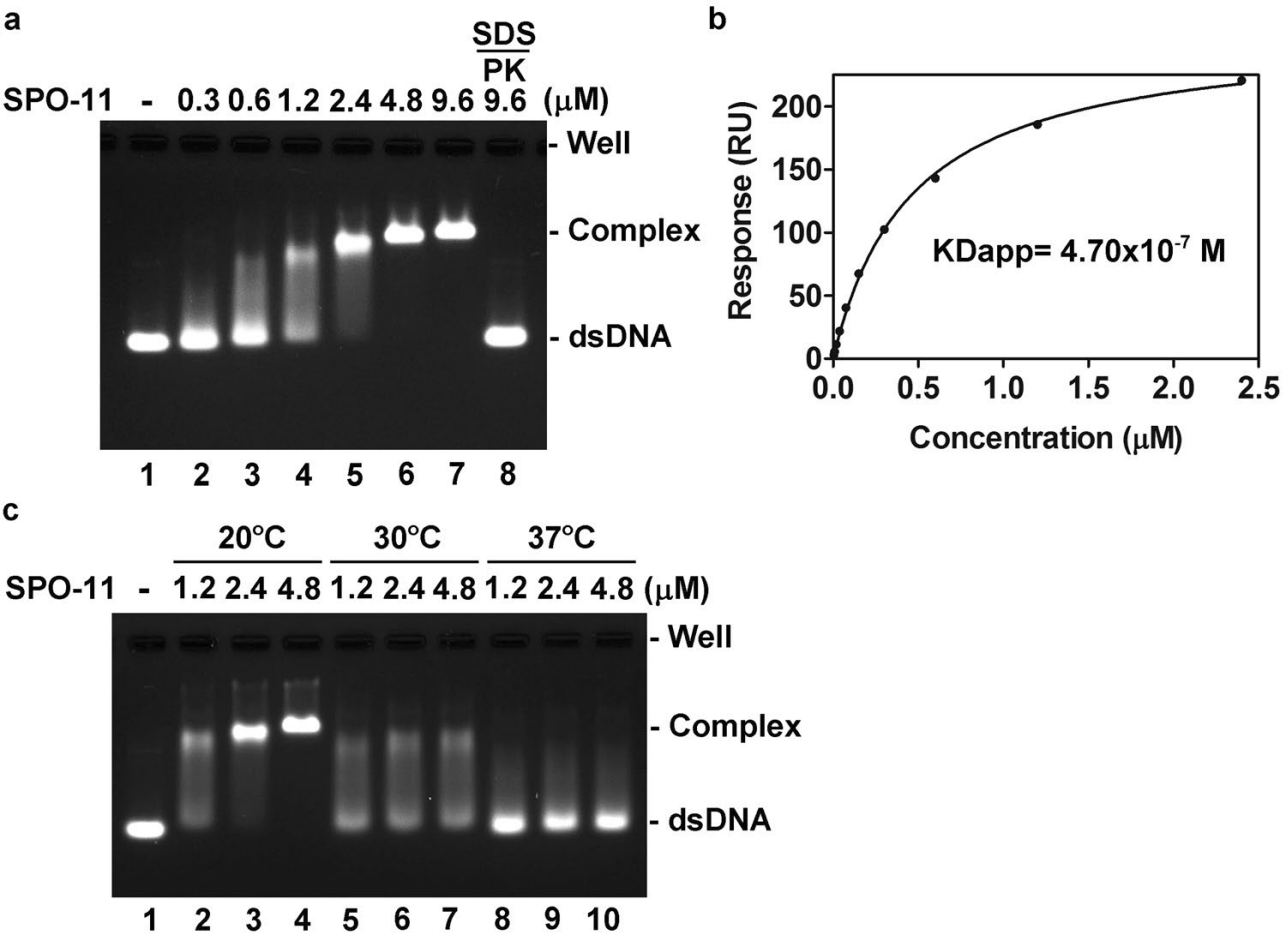
DNA-binding activity in SPO-11. (**a**) The indicated concentration of SPO-11 was incubated with a 100 bp dsDNA. In lane 8, the reaction mixture was treated with proteinase K (PK) and SDS to release the DNA from the nucleoprotein complex. (**b**) The interaction of indicated concentration of SPO-11 with an immobilized 100 bp dsDNA was analyzed by surface plasmon resonance. (**c**) DNA binding activity of SPO-11 is temperature sensitive.

To ask whether *C. elegans* SPO-11 binds DNA, the purified protein was incubated with a 100-bp duplex DNA followed by analysis in an agarose gel under non-denaturing conditions. SPO-11 protein bound the DNA substrate in a dosage-dependent manner **(**Fig. 3a **)**. Treatment of the nucleoprotein complex with SDS and proteinase K released unmodified DNA, indicative of the lack of nuclease activity **(**Fig. 3a, lane 8**)**. We then measured the equilibrium dissociation constant (KD) of DNA binding by SPO-11 by surface plasmon resonance (SPR). Briefly, a 5′-biotinylated duplex DNA was immobilized on streptavidin-coated SPR biosensor chip (SA chip), and purified SPO-11 protein was injected into the chip to determine the affinity constant between DNA and SPO-11. The results indicated that SPO-11 is a bona fide DNA-binding protein with an affinity of ~0.47 µM (Fig. 3b).

Because the optimal growth temperatures for nematodes range from 16 °C to 25 °C, we next determined the thermal stability of the SPO-11 DNA binding attribute. The results revealed the optimal temperature for SPO-11 to bind DNA as being 20 °C (Fig. 3c). Importantly, SPO-11 is highly susceptible to thermal denaturation as (1) pretreatment of the purified protein above 30 °C for 30 min abolished its DNA binding ability (Supplementary Fig. S1a); (2) circular dichroism analysis documented changes in the secondary structure of SPO-11 at higher temperatures (Supplementary Fig. S1b); and (3) the conformational changes of SPO-11 at elevated temperatures were readily detected by incubation with ANS (8-anilino-1-naphthalene sulfonate), a fluorescent probe that changes its fluorescent properties upon binding exposed hydrophobic residues in the target protein (Supplementary Fig. S1c).

### DNA binding properties of SPO-11

**Figure 4:**
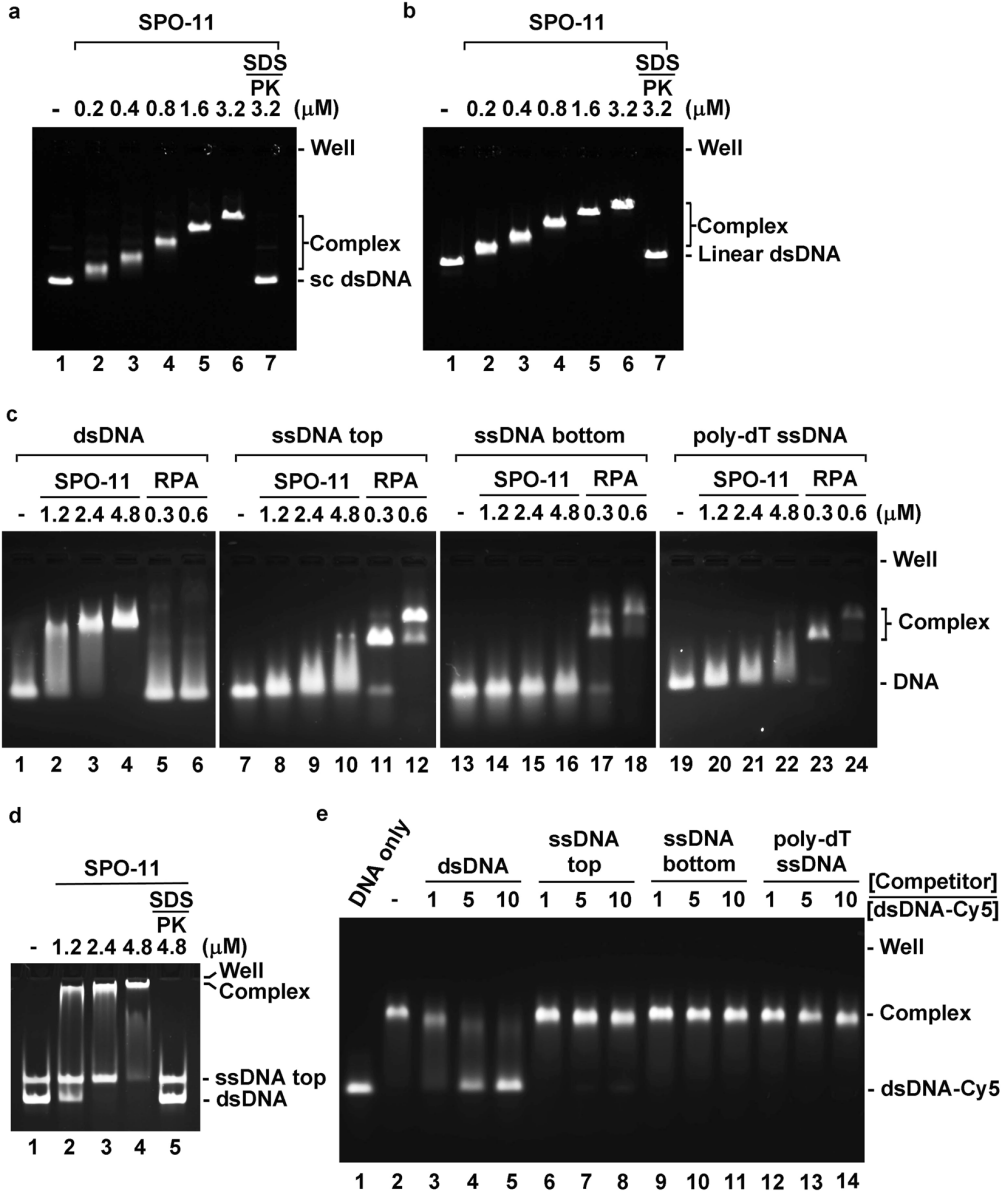
Preferential binding of dsDNA by SPO-11. Negatively supercoiled dsDNA (sc dsDNA) (**a**) or linear dsDNA (**b**) was used as substrate for SPO-11. (**c**) Binding of the 80 bp dsDNA, its constituent top and bottom ssDNA strands, and 80-mer poly-dT by SPO-11 was tested. The ssDNA binding protein RPA was included in the analysis. (**d**) The 80 bp dsDNA and top ssDNA strand were co-incubated with the indicated concentration of SPO-11. (**e**) Complex of SPO-11 and Cy5-labeled dsDNA was challenged with a 1, 5, or 10-fold molar amount of the indicated unlabeled DNA. Note that the agarose gels in panels a and b were stained with ethidium bromide; gels in panels c and d were stained with SYBR Gold to allow better detection of the ssDNA; DNA in the gel in panel e was detected by Cy5 fluorescence. In panels a, b, and d, PK denotes proteinase K.

We tested purified SPO-11 with negatively supercoiled and linear dsDNAs but found no significant binding preference for either substrate (Fig. 4a and b). Next, we compared the affinity of SPO-11 for ssDNA and dsDNA. While dsDNA binding reached saturation at 4.8 µM protein, there was only minimal binding of the three ssDNA substrates (top-strand, bottom-strand, and poly-dT) at this SPO-11 concentration (Fig. 4c). To further explore DNA binding preference, ssDNA and dsDNA were co-incubated with SPO-11 and then subject to the mobility shift assay, which clearly revealed a higher affinity for dsDNA (Fig. 4d). Moreover, non-labeled dsDNA was much more effective than non-labeled ssDNA in competing for SPO-11 pre-bound to fluorescently labeled dsDNA (Fig. 4e). Taken together, our results showed that SPO-11 possesses a significantly higher affinity for dsDNA.

**Figure 5:**
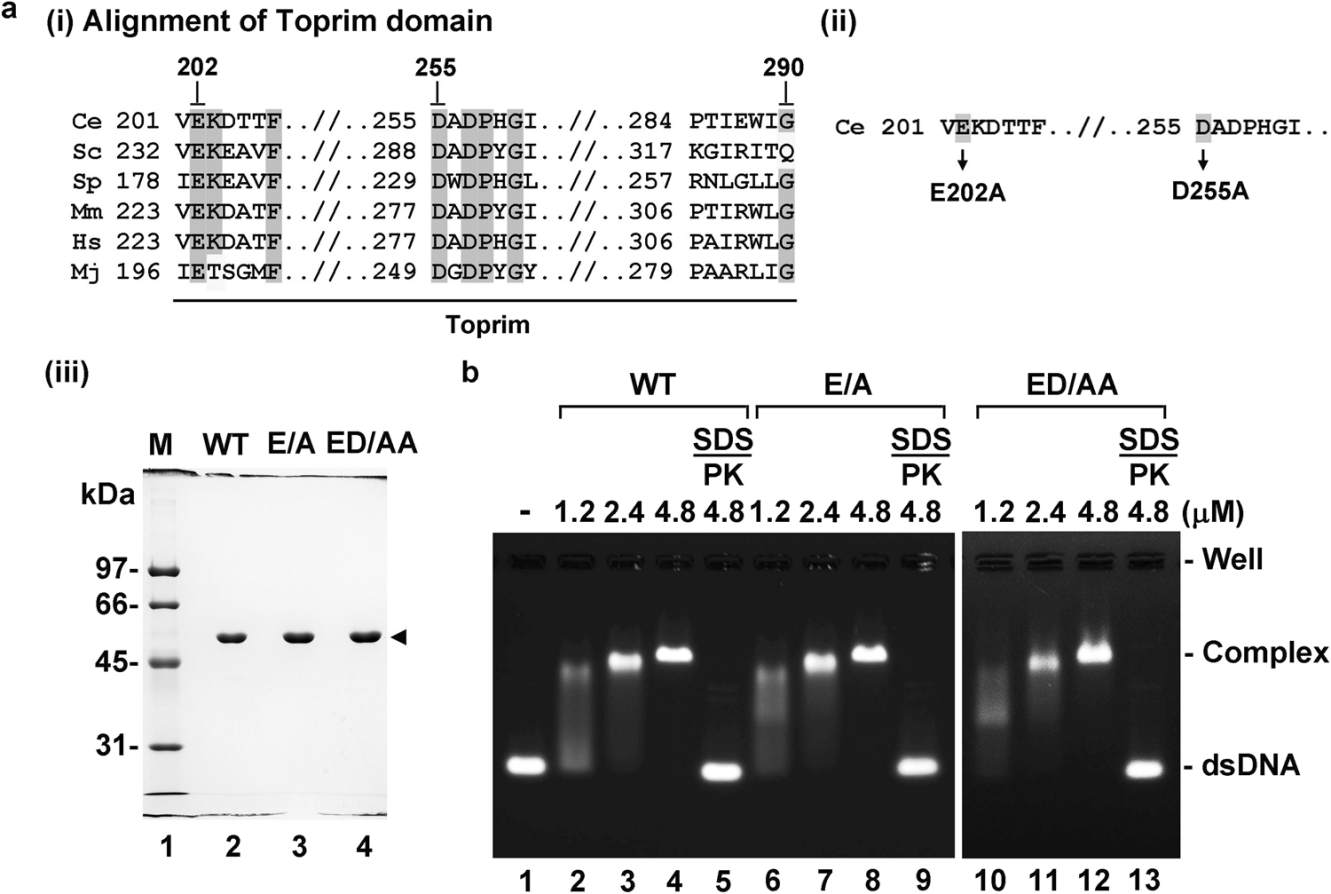
Lack of effect of magnesium on the DNA binding activity of SPO-11. (**a**) (i) Sequence alignment of the Toprim domain of SPO-11 from various species including *C.elegans* (Ce), *S. cerevisiae* (Sc), *S. pombe* (Sp), *M. musculus* (Mm), *H. sapiens* (Hs), and *M. janaschii* (Mj). (ii) *C. elegans* SPO-11 mutants generated in this study. (iii) Purified wild-type (WT), E202A (E/A) and E202A/D255A (ED/AA) SPO-11 proteins (1.5 μg each) were analyzed by SDS-PAGE. (**b**) The dsDNA binding activity of wild-type SPO-11 and mutants was analyzed with the 100 bp dsDNA in the presence of Mg^2+^. Symbol: PK, proteinase K.

Based on work done with *M. jannaschii* Topo VIA, it has been postulated that two evolutionarily conserved acidic residues, namely, glutamate-202 and aspartate-255, within the Toprim domain of SPO-11 play a role in enhancing DNA binding by coordinating Mg^2+^ (Fig. 5a, panel i ^25, 27^). To test this premise, we sought to determine whether DNA binding by *C. elegans* SPO-11 is responsive to Mg^2+^. The Mg^2+^ ion has little or no effect on enhancement of the DNA-binding ability of SPO-11 (Supplementary Fig. S2a). This result was further confirmed by monitoring the stability of the SPO-11-dsDNA complex by challenging it with increased salt with or without the presence of Mg^2+^. The stability of the protein-DNA complex remained the same regardless of whether Mg^2+^ was added or not (Supplementary Fig. S2b).

On the other hand, mutant variants with either a substitution of glutamate 202 to alanine (E202A) or a double substitution of glutamate 202 and aspartate 255 to alanine (E202A/D255A) were constructed and similarly expressed and purified as the wild-type protein and then tested for DNA binding (Fig. 5a, panels **ii & iii**). Importantly, the results revealed that both mutant proteins are just as proficient in DNA-binding as wild-type SPO-11, and that Mg^2+^ has no effect on DNA binding in all three cases (Fig. 5b, and Supplementary Fig. S3). In summary, unlike Topo VIA, Mg^2+^ does not significantly influence DNA-binding by SPO-11.

### SPO-11 lacks DNA cleavage activity

**Figure 6:**
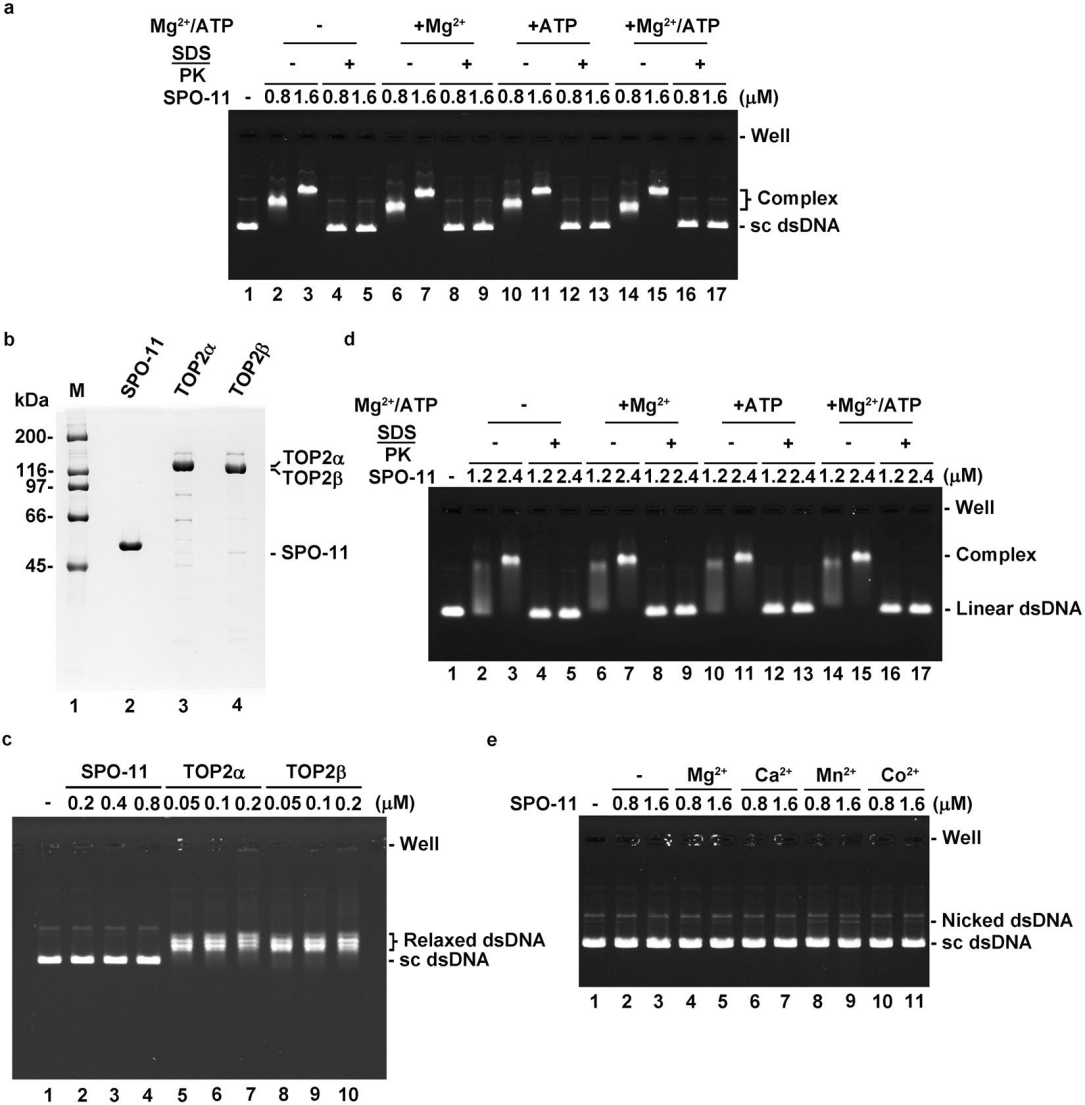
Lack of DNA cleavage activity in *C. elegans* SPO-11. (**a**) The indicated concentration of SPO-11 was incubated with negatively supercoiled dsDNA (sc dsDNA) with or without Mg^2+^, ATP, or Mg^2+^-ATP as indicated. DNA was released from nucleoprotein complexes by treatment with SDS and proteinase K (PK). (**b**) Purified SPO-11, human TOP2α, and TOP2β were analyzed by SDS-PAGE. (**c**) The indicated concentration of SPO-11, TOP2α, or TOP2β was incubated with negatively supercoiled dsDNA in the condition with the presence of Mg^2+^-ATP. (**d**) Linear dsDNA was used as the substrate to examine the DNA cleavage activity of SPO-11. (**e**) SPO-11 was examined for DNA cleavage activity with the indicated metal ion.

It remains unclear whether SPO-11 alone can catalyze DNA cleavage. We addressed this question by incubating SPO-11 with negatively supercoiled DNA under a variety of reaction conditions, followed by deproteinization of the reaction mixtures and analysis in an agarose gel^28^. We did not observe any DNA cleavage product regardless of whether Mg^2+^, ATP, or Mg^2+^-ATP was added or not (Fig. 6a). Type II topoisomerases human TOP2α and TOP2β were also included as the positive controls (Fig. 6b and c). Linear duplex DNA was also tested but, again, no DNA cleavage activity was observed (Fig. 6d). It has been reported that the inclusion of divalent metal ions can stimulate the activity of type II topoisomerase^7, 29–31^. However, no significant cleavage of supercoiled DNA was seen with Ca^2+^ or Co^2+^(Fig. 6e). We did observe a minor nicked product in the reaction with Mn^2+^ (Fig. 6e, lanes 8 & 9). To determine whether the DNA cleavage in these reactions reflected an intrinsic attribute of SPO-11 or might instead arise from a minor nuclease contamination, the catalytically dead mutant variants, SPO-11 Y119F and Y118F/Y119F, were similarly examined (Supplementary Fig. S4a). We note that both mutant variants are as proficient as the wild-type protein in DNA binding (Supplementary Fig. S4b). Importantly, the mutant protein preparations also generated the minor nicked product in the presence of Mn^2+^ (Supplementary Fig. S4c), indicating that this activity resulted from a minor nuclease contamination. Taken together, the results described herein provide strong evidence that SPO-11 lacks DNA cleavage activity.

### Characterization of the SPO-11 G290D mutant

**Figure 7:**
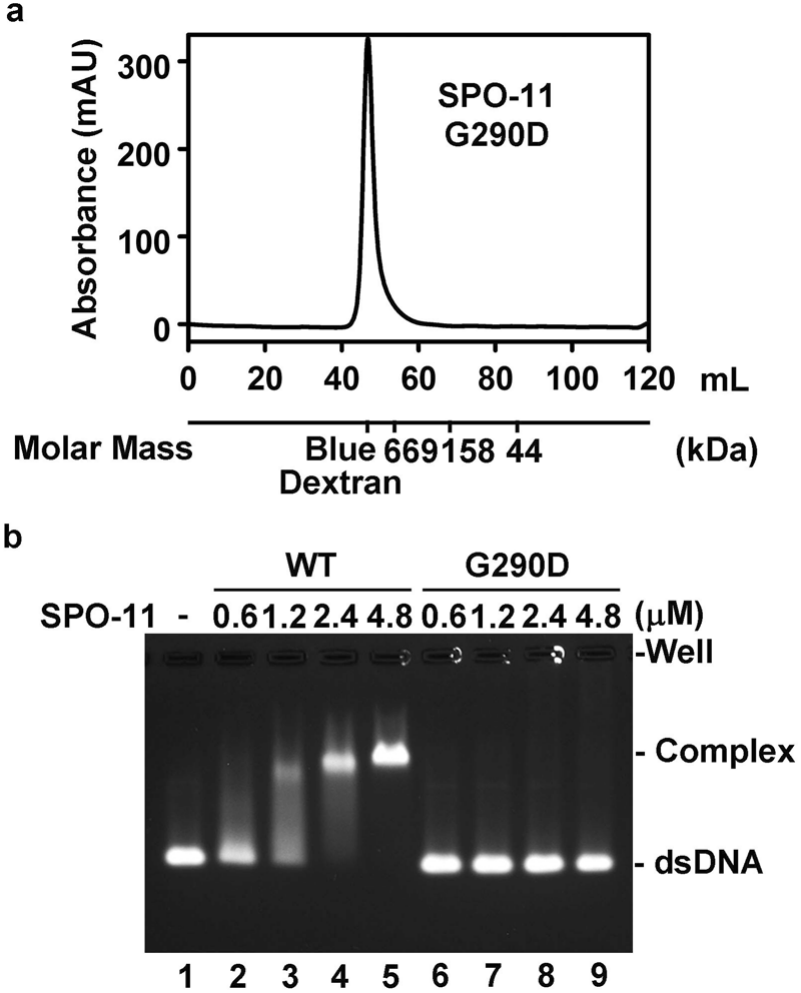
Characterization of the *C. elegans* SPO-11 G290D mutant. (**a**) Gel filtration analysis indicated that the SPO-11 G290D mutant protein is prone to forming a soluble aggregate. (**b**) The indicated concentration of wild-type SPO-11 (WT) or G290D mutant protein was tested for DNA binding using the 100 bp dsDNA as substrate.

The *me44* allele of *C. elegans* SPO-11 has been used extensively in genetic analyses as it behaves like a null allele^32–34^. This allele harbors a missense mutation that changes the highly conserved glycine-290 to aspartate (G290D)^32^. To determine the effect of the G290D mutation on SPO-11 activity, the mutant protein was expressed and purified. By gel filtration analysis, we found that the G290D protein is prone to forming a soluble aggregate (Fig. 7a). Importantly, we observed no DNA binding activity in this mutant protein (Fig. 7b).

## Discussion

Genetic studies from various model organisms have firmly implicated SPO-11 in DSB formation during meiosis^1–4^. However, only limited biochemical information on SPO-11 orthologs is available. Wahls and colleagues reported that recombinant *S. pombe* Spo11 protein solubilized from inclusion bodies and then renatured binds and cleaves supercoiled plasmid DNA^35^. Interestingly, purified trigger factor-tagged *A. thaliana* SPO11–1 protein binds DNA but lacks DNA cleavage activity^36^. In contrast, purified *O. sativa* Spo11-4 protein binds and cleaves duplex DNA^37^, although Spo11-4 is not required for the formation of DSBs in *O. sativa* meiosis^1, 37^. With such varied results, it remains unclear whether protein tagging affects the structural integrity of SPO11 and if the observed DNA cleavage activity in the resolubilized and renatured *S. pombe* Spo11 protein preparation might have originated from a nuclease contamination. Here, we have developed expression and purification procedures to obtain soluble, monodispersed, and untagged *C. elegans* SPO-11 protein. Our functional analyses documented a DNA-binding activity in SPO-11 that is specific for dsDNA. Importantly, we have provided evidence that SPO-11 alone lacks DNA cleavage activity. We have also demonstrated that the G290D mutation in the *spo-11 me44* allele affects SPO-11 protein conformation, leading to the formation of a soluble protein aggregate and loss of DNA binding activity. These findings can potentially help explain why the *spo-11* me44 behaves as a null allele in genetic analyses^32–34^.

Structural and functional analyses have implied that Mg^2+^ enhances the DNA binding activity of archaeal Topo VIA^25^. However, Mg^2+^ does not affect the DNA-binding affinity of *C. elegans* SPO-11. This observation was further supported by the lack of differential DNA binding affinity between wild-type and the E202A and E202A/D225A mutants predicted to be defective in Mg^2+^ binding. We have also confirmed that our purified SPO-11 is in a Mg^2+^-free form by inductively coupled plasma-mass spectrometry (ICP-MS; our unpublished results). In conclusion, there is a clear difference between archaeal Topo VIA and eukaryotic SPO-11. We note that mutating either of the two acidic residues equivalent to E202 and D225 of *C. elegans* SPO-11 in the *S. cerevisiae* Spo11 protein ablates DSB formation *in vivo* and results in a meiosis-defective phenotype^27^. Based on our results, we suggest that these conserved acidic residues may be involved in DNA cleavage activity and/or protein-protein interactions rather than affecting DNA binding activity. It will be interesting to test whether the *C. elegans spo-11* E202A and D255A mutants are also defective in meiotic DSB formation.

The crystal structure of archaeal Topo VIA reveals a dimeric structure in which the coordinated action of the two protein subunits in DNA cleavage generates a DSB^25, 26^. In contrast, a recent yeast two-hybrid and bimolecular fluorescent complementation (BiFc) study clearly demonstrated that SPO11-1 and SPO11-2, the two *A. thaliana* SPO11 isoforms responsible for meiotic DSBs, do not self-associate^22^. Furthermore, a meiotic Topo VIB-like (MTOPVIB) accessory factor is a prerequisite for the formation of a heterodimer of SPO11-1 and SPO11-2 in plants^3, 22, 23, 38^. Consistent with this observation, our biochemical and biophysical analyses have demonstrated that *C. elegans* SPO-11 is monomeric. Future investigation will determine whether SPO-11 forms a dimer on DNA and if other accessory factors are needed for or facilitate SPO-11 dimerization.

As the DNA cleavage activity of archaeal Topo VI requires the assembly of Topo VIA and Topo VIB subunits^24, 26, 28, 39^, it has long been speculated that a Topo VIB-like subunit exists to partner with SPO11. Recently, the Grelon and de Massy groups identified a meiotic Topo VIB-like partner of SPO11 in plant and mouse, respectively^4, 22, 23, 38^. These studies also showed that this Topo VIB-like factor interacts with SPO11 and is essential for DSB formation during meiosis^4, 22, 23, 38^. Results from our functional analyses also suggest that the putative topoisomerase activity of *C. elegans* SPO-11 is reliant on an unknown co-factor. Interestingly, no sequence homolog of Topo VIB has been found in *C. elegans* ^4, 22, 23^. Further investigations will be needed to identify the associated partners of SPO-11 in *C. elegans* and test them for the activation of SPO-11-mediated DSB formation. In this regard, the SPO-11 expression/purification procedures that we have developed and the SPO-11 mutants that we have generated should constitute a valuable resource in these future endeavors.

## Methods

### DNA substrates

To prepare substrates for DNA binding analysis, the 80-mer Oligo 1 (5′-TTATGTTCATTTTTTATATCCTTTACTTTATTTTCTCTGTTTATTCATTTACTTATTTTGTATTATCCTTATCTTATTTA-3′), Oligo 1 with its 3′ end labeled with Cy5, its exact complement Oligo 2 (5′-TAAATAAGATAAGGATAATACAAAATAAGTAAATGAATAAACAGAGAAAATAAAGTAAAGGATATAAAAAATGAACATAA-3′), and 80-mer poly-dT were synthesized and gel purified by Genomics BioSci & Tech. To prepare 80-mer duplex DNA, Oligo1 with or without 3′-Cy5 was incubated with Oligo 2 at 80 °C for 3 min, then at 65 °C for 30 min, followed by slow cooling to 23 °C for DNA annealing. The resulting duplex was purified from a 10% polyacrylamide gel by electro-elution and filter-dialyzed in a Centricon-10 concentrator (Millipore) at 4 °C into TE buffer (10 mM Tris-HCl, pH 8.0, and 0.5 mM EDTA). The 100-mer duplex (5′-AATATGATAGATAATGATAGTGATGAGGGACGTGGATCTCTTCTTACCTGCGGAGACGTCGAGGAGAACCCAGGACCAGGGGTACCTATGGCCTCCTCCG-3′) with or without 5′ biotin was synthesized by PCR and purified using the QIAquick^®^ PCR Purification kit (Qiagen). The supercoiled pBluescript II SK + plasmid was purified from *E. coli* by the plasmid midi kit (Qiagen). The linear form of pBluescript II SK + was prepared by digestion of the supercoiled DNA with EcoRV and purified using the QIAquick^®^ PCR Purification kit (Qiagen).

### Plasmids for *C. elegans* SPO-11 expression

*C. elegans* SPO-11 cDNA was inserted into the pET SUMO vector (Invitrogen) by TA cloning. SPO-11 E202A, SPO-11 E202A/D255A, SPO-11 G290D, SPO-11 Y119F, and SPO-11 Y118F/Y119F expression plasmids were generated by site-direct mutagenesis with pET SUMO-SPO-11 as the template. All the plasmids were sequenced to ensure there no unwanted mutation.

### *C. elegans* SPO-11 expression and purification

To express the amino-terminal (His)_6_- and SUMO-tagged SPO-11, pET SUMO-SPO-11 was transformed into the ArcticExpress (DE3) RIL strain harboring pG-Tf2, which harbors the chaperones GroES/GroEL and Trigger factor. The transformed cells were grown at 30 °C until the OD_600_ reached 0.6–0.8, at which time IPTG (final conc. 0.5 mM) and Tetracycline (final conc. 3 ng/ml) were added to induce the expression of SPO-11 and the chaperones, respectively. Cells were harvested by centrifugation after a 24-hr incubation at 12 °C. All the purification steps were carried out at 4 °C. For protein purification, 40 g cell pellet was suspended in 300 ml of T buffer (25 mM Tris-HCl, pH 7.5, 0.5 mM EDTA, 10% glycerol, 0.01% Igepal, 1 mM 2-mercaptoethanol) supplemented with 500 mM KCl, 2 mM Benzamidine, 0.2 mM PMSF, and 1 μg/ml of the following protease inhibitors: Aprotinin, Chymostatin, Leupeptin, and Pepstatin A, and then subjected to sonication. After ultracentrifugation (100,000 X g for 60 min), the clarified lysate was supplemented with 20 mM imidazole and incubated with 7 ml Ni^2+^ NTA-agarose (Qiagen) for 3 hr. After extensive washing with T buffer supplemented with 150 mM KCl and 50 mM imidazole, (His)_6_-SUMO-SPO-11 was eluted by T buffer supplemented with 150 mM KCl and 200 mM imidazole. The peak fractions were pooled and dialyzed against T buffer supplemented with 25 mM KCl and further applied to a 5 ml HiTrap^TM^ Heparin HP column (GE Healthcare), which was developed using a 60 ml linear gradient of 100–550 mM KCl. The (His)_6_-SUMO-SPO-11 fractions were recovered at ~400–475 mM KCl. The protein pool was diluted with 2 volumes of T buffer and further fractionated in a 1 ml HiTrap^TM^ Q HP column (GE Healthcare) using a 30 ml linear gradient of 200–700 mM KCl. The (His)_6_-SUMO-SPO-11 containing fractions (~400 mM KCl) were concentrated using a Centricon-10 concentrator (Millipore) and diluted with 2 volumes of T buffer. The SUMO protease Ulp1 (2 mg tagged SPO-11/μg protease) was then added, followed by an overnight incubation at 4 °C to remove the (His)_6_-SUMO tag. The reaction mixture was passed through Ni^2+^ NTA-agarose to separate SPO-11 from the freed tag. The now untagged SPO-11 was further purified by gel filtration in a HiLoad 16/600 Superdex 75 pg (GE Healthcare) column in T buffer supplemented with 300 mM KCl. The purified SPO-11 was concentrated to 1–5 mg/ml in a Centricon-10 concentrator and stored in 10 µl portions at −80 °C. All the SPO-11 mutants proteins were expressed and purified as described for the wild-type protein.

### Other protein reagents

Type II topoisomerases human TOP2α and TOP2β were purified as described^40, 41^.

### Electrophoretic mobility shift assay (EMSA)

Unless stated otherwise, the DNA binding experiments were carried out at 20 °C. Purified *C. elegans* SPO-11 or the indicated mutant was incubated with 300 nM of 100 bp dsDNA, 80 bp dsDNA, 80-mer ssDNA top strand (Oligo 1), ssDNA bottom strand (Oligo 2), 80-mer poly-dT, or 10 nM of pBluescript supercoiled or linear dsDNA, in 10 μl of reaction mixture (42.5 mM Tris-HCl, pH 7.5, 1 mM DTT, 115 mM KCl, 0.1 mg/ml BSA, 3% glycerol, and 0.15 mM EDTA) at 20 °C for 30 min. For competition analysis to determine the substrate-binding preference (see Fig. 4e), 2.4 μM SPO-11 was pre-incubated with 300 nM Cy5-labeled 80 bp dsDNA for 10 min. Then, 300, 1500, or 3000 nM of unlabeled 80 bp dsDNA, 80-mer ssDNA top strand or bottom strand, or 80-mer poly-dT was added, followed by a 20 min incubation. To determine the effect of Mg^2+^ on the stability of SPO-11-dsDNA complex (see Supplementary Fig. S2b), 1.8 μM SPO-11 was incubated with 300 nM 100 bp dsDNA with or without 10 mM MgCl_2_, and salt stringency increased from 100 to 1500 mM KCl as indicated. Reaction mixtures were resolved in 3% agarose gels for the short DNA substrates or 0.8% agarose gels for plasmid DNA substrates in TBE buffer (90 mM Tris-Boric acid, pH 8, and 2 mM EDTA) at 4 °C. The 100 bp dsDNA and plasmid DNA were stained with ethidium bromide and detected by Molecular imager^®^ Gel Doc^TM^ XR + station (Bio-Rad). The 80-mer duplex and single strand DNA were stained with SYBR^®^ Gold (Invitrogen^TM^) and detected in a BioSpectrum^®^ 810 imaging system (UVP).

### DNA cleavage analysis

For DNA cleavage experiments, SPO-11 was incubated with 300 nM 100 bp dsDNA or 10 nM supercoiled pBluescript in 10 μl of reaction mixture (42.5 mM Tris-HCl, pH 7.5, 115 mM KCl, 1 mM DTT, 0.1 mg/ml BSA, 3% glycerol, and 0.15 mM EDTA) in the absence or presence of 10 mM ATP, 10 mM MgCl_2_, or both. After incubation at 20 °C for 30 min, the reaction mixture was deproteinized by treatment with proteinase K (0.8 mg/ml) and SDS (0.1%) at 37 °C for 15 min. To test the effect of various divalent cations on the DNA cleavage activity of SPO-11, MgCl_2_ was replaced with 10 mM CaCl_2_, MnCl_2_ or CoCl_2_. Analysis of the reaction mixtures was as described above.

### Gel filtration analysis

SPO-11 or SPO-11 G290D was diluted to 0.5 ml T buffer supplemented with 300 mM KCl and analyzed by gel-filtration chromatography through a Superdex 75 10/300 GL column (GE Healthcare) or HiLoad 16/600 Superdex 200 pg (GE Healthcare), respectively. The protein size markers for calibration were also included.

### Analytical ultracentrifugation

The sedimentation velocity and sedimentation equilibrium of SPO-11 were analyzed in a Beckman-Coulter XL-A ultracentrifuge. Recombinant SPO-11 was diluted to the indicated concentrations in T buffer supplemented with 300 mM KCl. In the sedimentation velocity experiment, SPO-11 (9.2 μM) was loaded onto precooled standard double sector cells with an Epon charcoal-filled centerpiece. Centrifugation was performed at 4 °C and 50,000 rpm. The cells were scanned at 280 nm in a continuous mode, and the experimental data were analyzed by the Sedfit program (version 14.1). After ultracentrifugation, the protein sample was visually checked for clarity, and no indication of precipitation was found. In the sedimentation equilibrium experiment, SPO-11 (7.6 μM and 15.7 μM) were loaded onto a six-hole charcoal-filled Epon centerpiece individually, and absorbance profiles at 280 nm were monitored at rotor speeds of 11,000, 13,200, and 23,000 rpm at 4 °C until equilibrium was reached. The partial specific volume of SPO-11 and the buffer density were predicted using the SEDNTERP program. The equilibrium scans were analyzed with the software provided by Beckman-Coulter using a single ideal species mode^42^.

### Surface plasmon resonance

BIAcore T200 instrument (GE Healthcare) was used to determine the DNA binding affinity of recombinant SPO-11. A 100 bp biotinylated dsDNA was immobilized on a streptavidin sensor chip. Then, two-fold serially diluted SPO-11 starting at a concentration of 2.4 μM was injected into the flow cells at a flow rate of 30 μl/min at 20 °C in DNA binding buffer (42.5 mM Tris-HCl, pH 7.5, 115 mM KCl, 3% glycerol, 1 mM DTT, 0.15 mM EDTA, and 0.05% Tween 20). The sensor surface was regenerated with 1 M NaCl and 50 mM NaOH prior to a new injection. The obtained signals were subtracted from the reference channel that had not been coated with the DNA substrate. The results were plotted in a resonance unit against time sensorgram and the apparent dissociation constant was determined by steady-state affinity model using the Biaevaluation software (GE Healthcare).

## Electronic supplementary material

Supplementary Info
