# Supplementary material for: Functional characterization of the meiosis-specific DNA double-strand break inducing factor SPO-11 from C. elegans
Source: Sci Rep. 2017 May 24;7:2370. doi: 10.1038/s41598-017-02641-z (PMC5443798; doi:10.1038/s41598-017-02641-z)
Supplement: Supplementary file 1 — Supplementary Info [file 41598_2017_2641_MOESM1_ESM.pdf]

SUPPLEMENTARY INFORMATION

Functional characterization of the meiosis-specific DNA double-strand break inducing factor SPO-11 from *C. elegans*

Hsin-Yi Yeh[1], Sheng-Wei Lin[2], Yi-Chun Wu[3], Nei-Li Chan[4], and Peter Chi [1,2,*]

[1] Institute of Biochemical Sciences, National Taiwan University, NO. 1, Section 4, Roosevelt Road, Taipei 10617, Taiwan
[2] Institute of Biological Chemistry, Academia Sinica, 128 Academia Road, Section 2, Nankang, Taipei 11529, Taiwan
[3] Institute of Molecular and Cellular Biology and Department of Life Science, National Taiwan University, NO. 1, Section 4, Roosevelt Road, Taipei 10617, Taiwan
[4] Institute of Biochemistry and Molecular Biology, College of Medicine, National Taiwan University, NO. 1, Sec.1, Ren-Ai Rd., 100, Taipei, Taiwan

[*] To whom correspondence should be addressed:
Peter (Hung Yuan) Chi
Address:
Institute of Biochemical Sciences
College of Life Science
National Taiwan University
NO. 1, Sec. 4, Roosevelt Road, Taipei, 10617 Taiwan
Email: peterhchi@ntu.edu.tw
Phone: 886-2-23665573
Fax: 886-2-23635038

Supplementary Methods

Circular dichroism analysis

Circular dichroism (CD) spectrometry was performed to analyze the conformational change of SPO-11 in a spectropolarimeter (JascoJ-815) under constant N2 flush. The far-UV CD spectra were measured at 200-250 nm with a 1.0 bandwidth and a 0.2 nm resolution at a scan speed of 20 nm/min using a 1 mm path length quartz cuvette. The SPO-11 was diluted with K buffer (20 mM K_2HPO_4, pH 7.5, 0.5 mM EDTA, 10% glycerol, 0.01% Igepal, and 1 mM 2-mercaptoethanol) to the final 10 μM and pre-incubated at 20, 30, or 37°C for 30 min. Then, all CD spectra were collected at 20°C. The spectra were corrected for their respective buffer blanks, and five repetitive scans were averaged.

ANS fluorescence analysis

8-anilino-1-naphthalene sulfonate (ANS) fluorescence is used for analysis of protein structural properties. ANS exhibits intensive fluorescence upon interaction with the hydrophobic regions of a protein, but with low fluorescence intensity in solution. In order to analyze the conformational changes of SPO-11 at different temperatures, the ANS fluorescence was measured in a HITACHI F-4500 fluorescence spectrometer. SPO-11 was diluted with T buffer supplemented with 150 mM KCl to 0.3 mg/ml and pre-incubated with 0.1 mg/ml ANS at 20, 30, or 37°C for 30 min. Fluorescence of ANS was excited at 385 nm, and emission spectra were recorded between 400 and 600 nm at 20°C. The excitation and emission slits widths were fixed at 5 nm. For data evaluation, ANS emission spectra in buffer were subtracted from the corresponding ANS-protein spectra.

Supplementary Figures

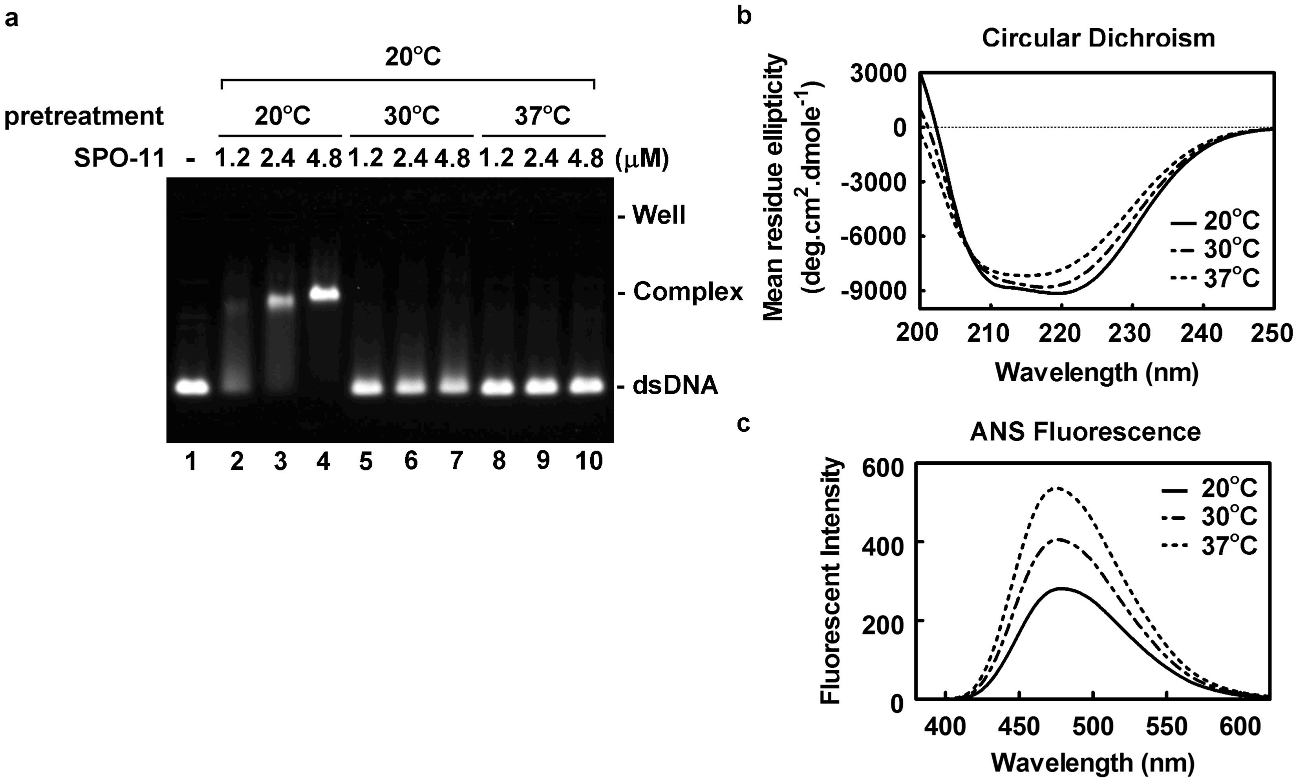

Supplementary Figure S1. SPO-11 is heat labile.
(**a**) SPO-11 was pretreated at the indicated temperature for 30 min and then tested for DNA binding at 20°C using the 100 bp dsDNA as substrate. (**b**) Circular dichroism spectra of SPO-11 at 20, 30, or 37°C. The spectra revealed a significant structural change at the elevated temperatures. (**c**) The thermal denaturation of SPO-11 was monitored by 8-anilino-1-naphthalene sulfonate (ANS) fluorescence.

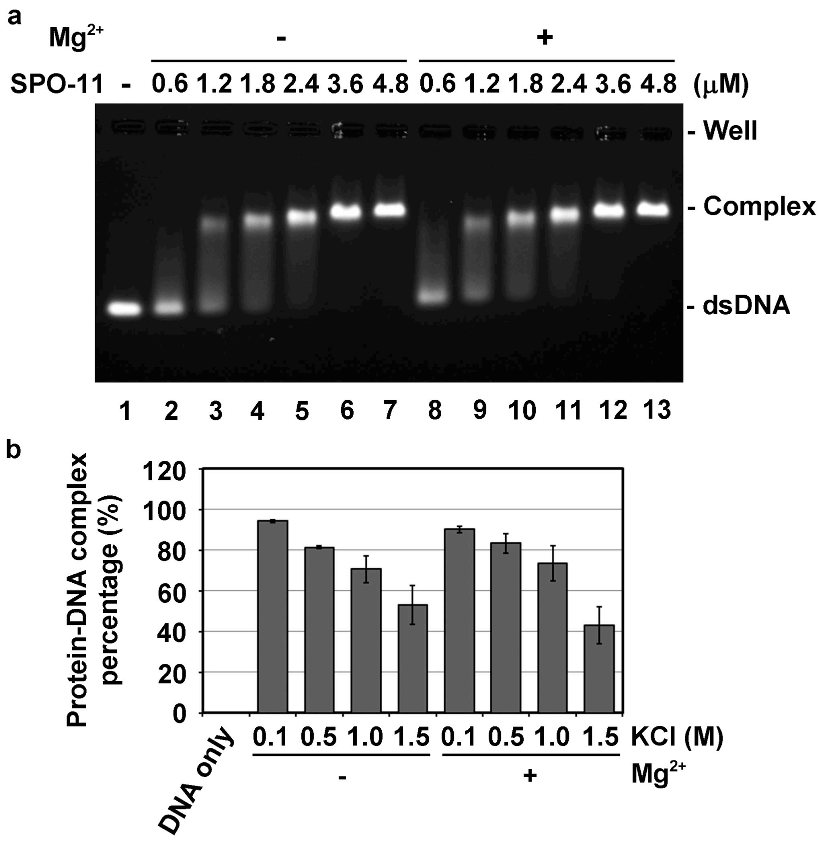

Supplementary Figure S2. The effect of magnesium on the DNA binding activity of SPO-11. (**a**) The indicated concentration of SPO-11 was incubated with the 100 bp dsDNA with or without 10 mM Mg^{2+}. (**b**) SPO-11-dsDNA complex was challenged by the indicated concentration of KCl with or without Mg^{2+} being present. Error bars represent the standard deviation (±SD) calculated based on at least three independent experiments.

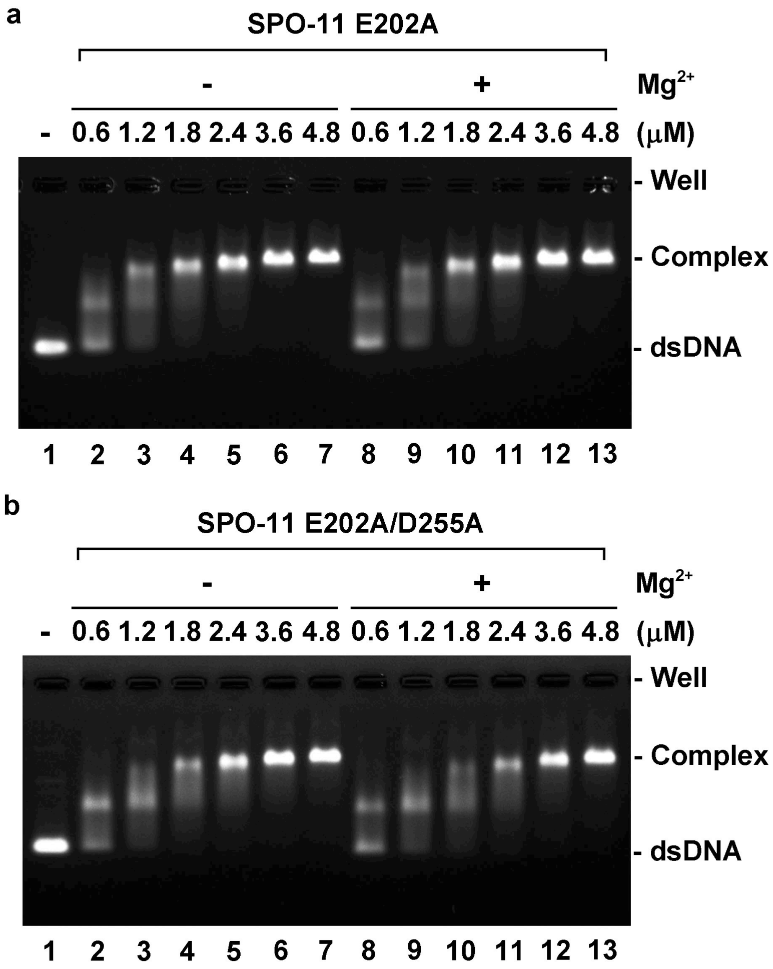

Supplementary Figure S3. Lack of effect of magnesium on the DNA binding activity of SPO-11 mutant proteins.

The indicated concentration of SPO-11 E202A (**a**) or E202A/D255A (**b**) was tested for binding of the 100 bp dsDNA with or without the presence of Mg^{2+}.

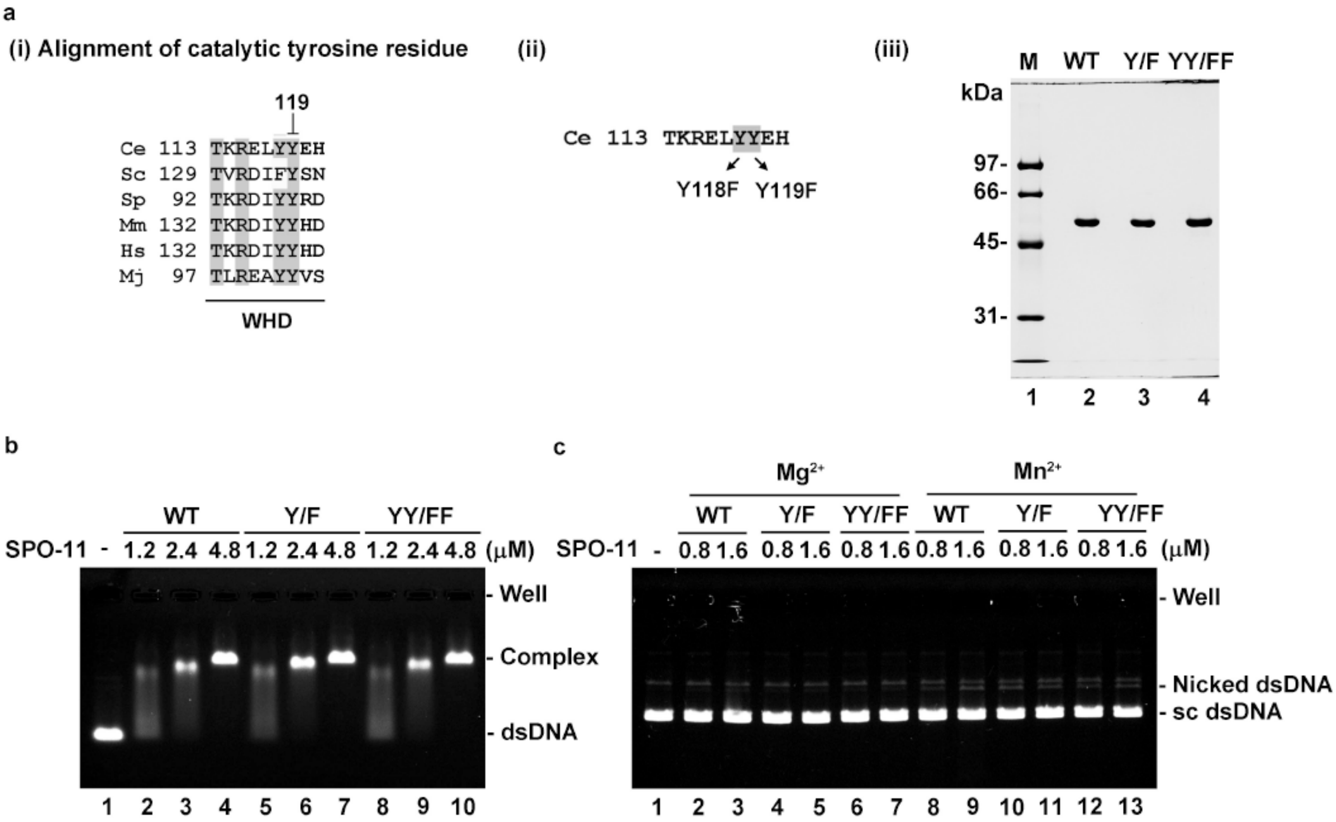

Supplementary Figure S4. Purification and biochemical characterization of SPO-11 catalytically dead mutant variants.
(**a**) (i) Sequence alignment of catalytic tyrosine residues within the winged-helix DNA-binding (WHD) domain of SPO-11 from various species including *C.elegans* (Ce), *S. cerevisiae* (Sc), *S. pombe* (Sp), *M. musculus* (Mm), *H. sapiens* (Hs), and *M. janaschii* (Mj). (ii) *Ce*SPO-11 mutants generated in this study. (iii) Purified wild-type (WT), Y119F (Y/F) and Y118F/Y119F (YY/FF) SPO-11 proteins (1.5 μg each) were analyzed by SDS-PAGE. (**b**) WT, YF, and YY/FF SPO-11 proteins were tested for DNA binding using the 100 bp dsDNA as substrate. (**c**) Wild-type and mutant SPO-11 proteins were tested for DNA cleavage with Mg^{2+} or Mn^{2+} present.
